# Non-Classical Complications of Adult-Onset Still’s Disease: A Multicenter Spanish Study

**DOI:** 10.3390/jcm14010285

**Published:** 2025-01-06

**Authors:** Javier Narváez, Judith Palacios-Olid, María Jesús García de Yebenes, Susana Holgado, Alejandro Olivé, Ivette Casafont-Solé, Santos Castañeda, Cristina Valero-Martínez, María Martín-López, Patricia E. Carreira, Maribel Mora-Limiñana, Laura Nuño-Nuño, Angel Robles-Marhuenda, Pilar Bernabeu, José Campos, Jenaro Graña, Vera Ortiz-Santamaria, Marisol Camacho-Lovillo, Carmen Vargas, Judith Sanchez-Manubens, Jordi Anton

**Affiliations:** 1 Department of Rheumatology, Hospital Universitario de Bellvitge, Bellvitge Biomedical Research Institute (IDIBELL), 08907 Barcelona, Spain; jpalacios.hv@gencat.cat (J.P.-O.); maribel.mora@sjd.es (M.M.-L.); 2 Instituto de Salud Musculoesquelética, 28045 Madrid, Spain; mjgarciadeyebenes@inmusc.eu; 3 Hospital Universitari Germans Trias i Pujol, 08916 Badalona, Spain; sholgado.germanstrias@gencat.cat (S.H.); olivealejandro8@gmail.com (A.O.); icasafont.germanstrias@gencat.cat (I.C.-S.); 4 Hospital Universitario de La Princesa, IIS-Princesa, Cátedra UAM-Roche, Universidad Autónoma de Madrid, 28006 Madrid, Spain; santos.castaneda@salud.madrid.org (S.C.); cristina.valmart@gmail.com (C.V.-M.); 5 Hospital Universitario 12 de Octubre, 28041 Madrid, Spain; mmartinlopez@salud.madrid.org (M.M.-L.); carreira@h12o.es (P.E.C.); 6 Hospital Universitario La Paz, 28046 Madrid, Spain; laura.nuno@salud.madrid.org (L.N.-N.); aroblesmarhuenda@gmail.com (A.R.-M.); 7 Hospital General Universitario de Alicante, 03010 Alicante, Spain; bernabeu_pil@gva.es; 8 Hospital Universitario Puerta de Hierro, 28222 Madrid, Spain; jose.campos@salud.madrid.org; 9 Complejo Hospitalario Universitario de A Coruña, 15006 A Coruña, Spain; genaro.grana.gil@sergas.es; 10 Hospital General de Granollers, 08402 Barcelona, Spain; vortiz@fhag.es; 11 Hospital Universitario Virgen del Rocío, 41013 Sevilla, Spain; soledad.camacho.sspa@juntadeandalucia.es; 12 Hospital Universitario Virgen Macarena, 41009 Sevilla, Spain; cvargaslebron@hotmail.com; 13 Hospital Universitari Parc Taulí, 08208 Sabadell, Spain; jsanchez@tauli.cat; 14 Department of Pediatric Rheumatology, Institut de Recerca Sant Joan de Déu, Hospital Sant Joan de Déu, Universitat de Barcelona, 08950 Barcelona, Spain; janton@ub.edu

**Keywords:** adult-onset still disease, clinical manifestations, prognostic

## Abstract

**Objective:** To investigate the prevalence and clinical spectrum of atypical or non-classical complications in adult-onset Still’s disease (AOSD) beyond macrophage activation syndrome (MAS) and to identify factors linked to their occurrence. **Methods:** Multicenter cross-sectional study of AODS cases included in the Spanish registry on Still’s disease. **Results:** This study included 107 patients (67% women), of whom 64 (59.8%) developed non-classical complications. These include macrophage activation syndrome in 9.5%, atypical skin manifestations in 38.8%, cardiac involvement in 22.7% (comprising pericarditis, myocarditis, pulmonary arterial hypertension, and noninfectious endocarditis), pleuritis in 28.9%, transient pulmonary infiltrates in 4%, significant headache in 14.1%, lower abdominal pain with evidence of peritonitis in 8.4%, and secondary amyloidosis in 0.9%. In the multivariate logistic regression analysis, lymphadenopathy (OR 2.85, 95% CI 1.03–7.91, *p* = 0.044) and the systemic score system (SSC) index (OR 1.86, 95% CI 1.29–2.69, *p* = 0.001) were independently associated with the development of non-classical clinical manifestations. In contrast, typical exanthema was associated with a reduced risk of these complications (OR 0.32, 95% CI 0.11–0.95, *p* = 0.041). **Conclusions:** In addition to the typical clinical manifestations and MAS, a significant proportion of patients with AOSD develop uncommon complications, some of which can be potentially life-threatening. These should be considered in the evaluation and follow-up of patients. Early recognition and prompt management are crucial to significantly reduce morbidity and mortality.

## 1. Introduction

Adult-onset Still’s disease (AOSD) is a rare systemic inflammatory disease characterized typically by high fever, evanescent skin rash, arthritis, leukocytosis, and hyperferritinemia, which are the hallmarks of the disease [1,2,3,4,5]. Other common manifestations include odynophagia, myalgias, hepatic involvement (elevated liver enzymes with or without hepatomegaly), lymphadenopathy, and splenomegaly [1,2,3,4,5].

Increasing evidence highlights the significant heterogeneity of AOSD in terms of clinical presentation, severity, and progression. For reasons yet unclear, a subset of patients develop severe, potentially life-threatening complications, including macrophage activation syndrome (MAS), atypical skin manifestations, and hematological, cardiac, pulmonary, renal, or neurological involvement [1,2,3,4,5,6].

This study aimed to investigate the prevalence and clinical spectrum of the non-classical complications in AOSD patients beyond MAS and to identify factors associated with their occurrence.

## 2. Methods

This was a multicenter study of prevalent cases from a national Spanish investigation of Still’s disease, including systemic juvenile idiopathic arthritis (sJIA) and adult-onset Still’s disease (AOSD). Our analysis focused exclusively on AOSD patients, covering both prevalent and incident cases managed in the rheumatology or internal medicine departments of tertiary hospitals across Spain. Patients were eligible if they had a confirmed diagnosis of AOSD, regardless of the diagnostic criteria applied, and had been followed for at least one year at the participating hospitals. To ensure the broad representativeness of the sample, at least 10 centers from different regions of Spain were required to participate, and the recruitment period was set at 10 months.

A retrospective analysis of medical records was performed in accordance with a predefined data collection protocol. The methodological and general characteristics of the JIA-Still SERPE registry have been published previously [7]. Patients were excluded if more than 50% of the required data from their clinical records were unavailable. For the purpose of this study, the following data were collected: (A) epidemiological characteristics, including age, sex, onset forms, and disease progression; (B) diagnostic criteria (Cush [8], Yamaguchi [9], and Fautrel [10]); (C) clinical variables at onset and during disease evolution; (D) quality of life and functional capacity assessed using the EuroQoL score and the Health Assessment Questionnaire (HAQ), respectively); (E) laboratory parameters (ESR, C-reactive protein, hemoglobin, leukocytes, neutrophils, platelets, ferritin, and comprehensive liver function tests) and imaging findings (radiography, joint ultrasonography, echocardiography, and computed tomography [CT]); and (F) sequelae or chronic damage attributable to the disease or its treatment.

To standardize data collection and ensure consistency, a detailed form and a codebook were created, specifying variable definitions and validation rules to guide researchers during the analysis phase.

Patients, or legal guardians in the case of minors, signed an informed consent form for the collection of their data. The data were recorded on a dedicated online platform developed for this study. The data were confidentially processed in accordance with European norms. This study was conducted following the principles of the Declaration of Helsinki and the International Conference for Harmonisation of Technical Requirements for Pharmaceuticals for Human Use.

Statistical analysis. The results are expressed as the mean ± standard deviation (SD) or as the median (interquartile range [IQR], 25th–75th percentile), as appropriate for continuous variables, whereas categorical variables are presented as frequencies and percentages. The prevalence of atypical manifestations and their 95% confidence intervals (CIs) were calculated assuming a Poisson distribution. The numerical variables were compared using the Student’s *t*-test or Mann–Whitney *U* test, depending on the normality of their distribution, while categorical variables were compared using the chi-squared test or Fisher’s exact test.

Finally, bivariate and multivariate logistic regression models were applied, with the dependent variable defined as the presence of atypical or non-classical complications. Independent variables included sociodemographic data, selected baseline clinical characteristics (based on the results of the comparative study), type of disease course, laboratory parameters, and systemic score system index values. Variables with clinical relevance or a *p*-value < 0.25 in the bivariate analysis were included in the multivariate models. Statistical significance was set at *p* < 0.05.

## 3. Results

The cohort included 107 patients from 14 centers across various autonomous communities in Spain. Table 1 summarizes their general characteristics and main clinical and laboratory data. Most patients were women (67%), with a median age at diagnosis of 40.6 years (IQR 30.2–53.8). The median interval between symptom onset and diagnosis was 0.13 years (IQR 0.06–0.44), and 30.2% of the patients reported a family history of related conditions. The Yamaguchi diagnostic criteria were the most frequently fulfilled (85%), followed by Cush (58.9%) and Fautrel (54.2%).

Fever was present in all patients (100%), arthralgia in 99%, arthritis in 68.9%, and typical exanthema in 82.6%. Other common features included odynophagia in 78.4%, constitutional syndrome in 44.4%, hepatic involvement in 29.5%, lymphadenopathy in 43.4%, and splenomegaly in 28.4%.

The erythrocyte sedimentation rate (ESR) was elevated (≥30 mm/h) in 92.8% of patients. The mean ferritin level was 6053 ng/dL (SD ± 9779), with 56.5% of patients showing levels ≥1500 ng/dL. Leukocytosis exceeding 15,000/mm^3^ was observed in 43.7% of cases. Elevated liver enzyme levels (ALT, AST, or GGT > 40 U/L) were detected in over half of the patients.

Glucocorticoids were used in 63.6% of patients, and 58.8% required biologic agents. The disease course was mono-episodic in 27.4% of the patients, poly-episodic in 27.4%, and persistent in 45.3%.

### 3.1. Non-Classical Clinical Manifestations

Among the 107 patients, 64 (59.8%; 95% CI: 46.06 to 76.38) presented with one or more of the following manifestations (see Table 2):

- MAS, defined by the Ravelli criteria or confirmed by pathological findings, was identified in 10 out of 105 patients, with a prevalence of 9.5% (95% CI: 4.57 to 17.51). No patients with other severe hematological complications, such as thrombotic thrombocytopenic purpura or disseminated intravascular coagulopathy, were identified.
- Atypical skin manifestations evaluated by a dermatologist were observed in 38 of 98 patients (38.8%, 95% CI: 27.44 to 53.22). The most common lesions included persistent pruritic papules and plaques with a linear configuration resembling flagellate erythema and urticaria-like eruptions.
- Cardiac complications were identified in 22 of 97 patients (22.7%, 95% CI 14.21 to 34.34), including 18 cases of pericardial disease (18.5%, 95% CI 11.0 to 29.33) confirmed by transthoracic echocardiogram and/or thoracic CT; 4 cases of myocarditis (4.1%, 95% CI 1.12 to 10.56), defined by compatible symptoms, elevated troponin levels, and nonspecific ECG abnormalities, and supported by echocardiographic findings; 6 cases of suspected pulmonary arterial hypertension (PAH) (6.2%, 95% CI 2.27 to 13.46) identified by transthoracic echocardiogram; and 1 case of inflammatory valvular involvement (1%, 95% CI 0.03 to 5.74) confirmed by both transthoracic and transesophageal echocardiograms.
- Pleural and pulmonary involvement: pleural disease, confirmed by chest X-ray and/or thoracic CT, was identified in 28 of 97 patients (28.9%, 95% CI 19.18 to 41.72). Transient pulmonary infiltrates, excluding infection and acute pulmonary edema, were observed in 4 of 100 patients (4%, 95% CI 1.09 to 10.24) on chest X-ray and/or high-resolution thoracic CT.
- Significant headache was reported in 13 of 92 patients (14.1%, 95% CI 7.52 to 24.16), with some cases objectively confirmed as aseptic meningitis through lumbar puncture with cerebrospinal fluid (CSF) analysis and cranial CT or MRI.
- Peritonitis, confirmed by imaging revealing peritoneal effusion (ultrasound and/or abdominal CT), was observed in 9 of the 107 patients (8.4%, 95% CI 3.85 to 15.97). The proportion of patients with any form of serositis (pleuritis, pericarditis, or peritonitis) in our cohort was 30.9% (30/97, 95% CI 22.60 to 40.70).
- Secondary amyloidosis, confirmed by biopsy, was identified in 1 of 104 patients (0.9%, 95% CI 0.02 to 5.36).

Chronic damage, defined as radiological (X-ray and/or joint ultrasonography) or functional impairment, was observed in 14% of cases (15/107; 95% CI 8.68 to 21.84). The frequency of other disease- or treatment-related complications/chronic damage is detailed in Table 3.

### 3.2. Predictors of Development of Non-Classical Clinical Manifestations

In the comparative study (Table 4), patients with complications had a significantly greater incidence of lymphadenopathy (52.4% vs. 27.8%; *p* = 0.018) and, as expected, higher scores on the systemic score system index (6.6 vs. 5.3; *p* = 0.0002). Additionally, these patients were more likely to present with a chronic clinical course, elevated ferritin levels, liver function test abnormalities, a higher prevalence of hepatomegaly, and an increased need for high-dose steroids and biological therapy, although these differences did not reach statistical significance.

In the multivariate logistic regression analysis (Table 5), lymphadenopathy (OR 2.85, 95% CI 1.03–7.91, *p* = 0.044) and the systemic score system (SSC) index (OR 1.86, 95% CI 1.29–2.69, *p* = 0.001) were independently associated with the development of non-classical clinical manifestations. In contrast, typical exanthema was associated with a reduced risk of these complications (OR 0.32, 95% CI 0.11–0.95, *p* = 0.041).

## 4. Discussion

In addition to typical manifestations and MAS, more than half of patients with AOSD develop uncommon clinical manifestations that warrant consideration, particularly during evaluation and follow-up. Early recognition and timely intervention are essential for reducing morbidity and mortality.

In addition to the classic evanescent rash, atypical cutaneous manifestations have been reported in up to 14% of patients with AOSD [11], reaching 38.8% in our series. The most common and representative lesions are persistent pruritic papules and plaques, which typically range in color from erythematous to brown or violaceous [11,12]. They may present with scales or crusts and are predominantly located on the back, upper chest, abdomen, and extensor surfaces of the extremities. A linear distribution is frequently observed, likely associated with the Koebner phenomenon, resulting in a flagellate, erythema-like appearance. Histologically, these lesions exhibit a distinctive pattern characterized by singly or clustered dyskeratotic/necrotic keratinocytes in the upper epidermis, coupled with a perivascular inflammatory infiltrate in the upper and middle dermis [11]. Less commonly, other lesions such as urticaria and urticaria-like eruptions, may occur [11,12]. These manifestations may appear at any stage of the disease, most commonly in patients with persistent and severe disease activity. Recognizing this clinical variant is essential for the early diagnosis of AOSD, as it may indicate persistent disease activity and the need for more intensive treatment [11,12].

Cardiac involvement is one of the most common visceral complications in AOSD, occasionally representing the initial presentation of the disease and one of the main causes of early mortality associated with it [1,2,3,4,5,6,13]. In our series, its prevalence was 22.7%, which is comparable to the 29% reported by Bodard et al. [13]. The most common cardiac complication is pericarditis, with or without tamponade, the prevalence of which in previous studies ranges between 10% and 37% of cases [1,2,3,4,5,6,14] (11.5% of AOSD patients included in the international AIDA Network Still’s disease registry) [15]. Notably, no specific prevalence for tamponade has been reported to date. Following pericarditis, myocarditis is the second most frequent cardiac complication, with an estimated prevalence of 7% in the literature [16,17], compared with the 4.1% observed in our cohort. Pericarditis is frequently associated (myopericarditis), occurring in 54% of cases [16,17]. Myocarditis typically manifests early, within the first year of disease progression in 80% of patients, and it is present at the time of AOSD diagnosis in 54% of patients [17]. Patients with AOSD-associated myocarditis are generally younger and predominantly male (75%) [16,17]. Common symptoms include fever, chest pain, dyspnea, and tachycardia. Therefore, AOSD should be considered in cases of acute febrile myocarditis.

Other less common complications include PAH and noninfectious endocarditis. The prevalence of PAH, estimated at 4.8% in AOSD patients over 33 years of follow-up [18], is comparable to the 6.2% observed in our cohort. Valvular involvement has rarely been described in the literature [19]. The underlying pathophysiological mechanisms remain unknown, but corticosteroids appear to be effective. Histological analysis, when conducted, identified a fibrinoid or fibrinous-clot component.

In our series, the prevalence rates of pleuritis and parenchymal lung involvement were 28.9% and 4%, respectively. Pleuritis is the most common pulmonary manifestation of AOSD, with an estimated clinical prevalence ranging from 10% to 28% [1,2,3,4,5,6,14] (14.6% among AOSD patients included in the international AIDA disease registry) [15]. It is associated with severe disease activity and is recognized as an unfavorable prognostic factor in AOSD patients. The presence of serositis at diagnosis—either pericarditis in some cases [20] or pleuritis in others [21]—has been linked to a greater likelihood of requiring biological therapy during follow-up. Some authors advocate for the earlier use of biologics in such cases [21]. Interstitial pneumopathy has been reported in 5.3% of AOSD cases [22,23], presenting in two distinct forms. (1) The first form is acute respiratory distress syndrome (ARDS), which is observed in 40% of cases and occurs exclusively in patients with a systemic profile as an early complication of AOSD (either at onset or within the first year). This form is characterized by symptoms such as dyspnea, cough, and bilateral infiltrates, sometimes accompanied by diffuse alveolar hemorrhage [22,23]. (2) The second form is interstitial lung disease (ILD) without ARDS, which is observed in 60% of patients, with a radiological pattern of organizing pneumonia, nonspecific interstitial pneumonia (NSIP), or unclassifiable interstitial pneumopathy. In these cases, pulmonary involvement was the initial manifestation in half of the patients, with cough and dyspnea being the most common symptoms (72% and 44%, respectively) [22,23]. Auscultatory abnormalities are rare. The vast majority of these patients had the systemic form of AOSD.

Headache is a relatively common symptom at disease onset or during relapse, reported in 7% to 12% of cases in the literature [23,24,25] and observed in 14.1% of our patients. Both the central and peripheral nervous systems can be affected. Aseptic meningitis is the most frequent neurological manifestation, accounting for 64.3% of all cases of nervous system involvement [24,25,26]. Although rare, other neurological complications include meningoencephalitis, seizures, ischemic and hemorrhagic strokes, Miller Fisher syndrome, demyelinating encephalopathy, cranial nerve palsies, symmetrical peripheral sensory abnormalities, and sensorineural hearing loss [24,25,26]. Although neurological involvement is generally considered a late-stage complication, it has occasionally been reported as the initial presentation of the disease, particularly in cases of aseptic meningitis.

Lower abdominal pain is also not uncommon in AOSD, as it has been reported in up to 13% of patients [27] (11.1% in the AIDA Network registry) [15]. It has been attributed to hepatosplenomegaly, mesenteric adenitis, and serous peritonitis, although the underlying cause is rarely documented. In our series, peritonitis was identified through imaging techniques in 8.4% of patients.

AA amyloidosis (AAA) is a rare complication of AOSD that typically develops during long-term follow-up as a consequence of persistent or recurrent inflammation due to suboptimal disease control. This complication has been described primarily in case reports of chronic refractory articular AOSD with prolonged disease courses and is often associated with joint destruction [28]. Its prevalence remains unknown. In our cohort, secondary amyloidosis was confirmed in 1% of patients, reflecting its rarity in the context of AOSD.

A key challenge in evaluating patients with AOSD is identifying predictors at the time of diagnosis that can help determine those at increased risk of developing atypical or non-classical complications, some of which are potentially life-threatening. Identifying reliable predictors is particularly crucial, especially now that effective and safe biologic agents (such as canakinumab, anakinra, tocilizumab, or anti-TNF agents) are available. Defining optimal treatment strategies could minimize the cumulative glucocorticoid burden. In our series, patients with complications had a significantly greater incidence of lymphadenopathy and higher systemic score system index values.

Limited information is available in this area. In our study, lymphadenopathy and the systemic score system index were identified as factors associated with the development of non-classical clinical manifestations. A plausible hypothesis is that lymphadenopathy reflects heightened systemic inflammation and immune activation driven by excessive cytokine production (e.g., IL-1β, IL-6, IL-18) [1,2,3,4,5,6,29]. This inflammatory activity could predispose patients to non-classical complications by exacerbating immune dysregulation and promoting multi-organ involvement. Additionally, it may indicate more severe disease phenotypes associated with atypical manifestations. Higher systemic score values at the time of diagnosis have emerged as the most consistently validated predictive factor. In a recent study involving 597 patients with AOSD included in the Gruppo Italiano di Ricerca in Reumatologia Clinica e Sperimentale (GIRRCS) AOSD-Study Group and the Autoinflammatory Disease Alliance Network Still Disease Registry, a systemic score ≥ 7 was shown to significantly predict the likelihood of a life-threatening disease course (OR 3.36) [30]. An analysis of the clinical relevance of individual systemic score components revealed that both liver involvement and lung disease were significant predictors of life-threatening evolution. Wahbi et al. [31] recently identified catastrophic adult-onset Still’s disease (CAOSD) as a distinct subset defined by life-threatening organ complications requiring intensive care. The major complications reported included cardiac involvement (myocarditis, tamponade), pulmonary distress (including ARDS), and coagulopathies such as disseminated intravascular coagulation. These severe complications predominantly occur during the initial disease flare, resulting in rapid onset and marked systemic inflammation. Compared with 41 AOSD control patients without organ failure, three independent predictors of life-threatening complications were identified via multivariate analysis: the absence of arthralgia, younger age, and a shorter interval between fever onset and hospitalization [31]. In another French series, 33% of patients presented with organ complications [32]. Fever > 39.5 °C was identified as predictive of monocyclic AOSD, whereas arthritis and thrombocytopenia were associated with chronic and complicated AOSD, respectively [32]. However, this association may be confounded, as the most prevalent complication in these patients was MAS, a condition in which thrombocytopenia is almost invariably present. Younger patients were found to have the highest risk of resistance to first-line treatments.

When interpreting the study results, several potential limitations should be considered: (1) its observational and retrospective design, which entails a risk of information bias due to inaccurate data recording and the potential underestimation of certain complications; (2) the limited representativeness of the sample; (3) the inclusion of patients who did not uniformly fulfill a single diagnostic criterion; (4) potential selection biases resulting from nonuniform assessment criteria; and (5) incomplete data collection on administered biologic therapies and patient follow-up, as this study was limited to a cross-sectional phase, restricting our ability to provide reliable information on these aspects. Despite these limitations, this study is the first to specifically evaluate the frequency of these atypical manifestations of AOSD within a relatively large cohort of patients with this rare disease, reflecting real-world clinical practice. It constitutes the largest cohort studied in this context and provides valuable, high-quality information on the prevalence of these complications.

In conclusion, beyond the typical clinical manifestations and MAS, a significant proportion of patients with AOSD experience uncommon complications, some of which may be life-threatening. These complications should be carefully considered, particularly during patient evaluation and follow-up. Early recognition and timely management are essential to reducing morbidity and mortality. Future studies are needed to investigate whether these non-classical complications observed in AOSD might also occur in patients with sJIA, given the growing recognition of their shared disease spectrum [33].

## Figures and Tables

**Table 1:** The main characteristics of the 107 patients with AOSD.

Number of Patients *	*n* = 107
**Demographic characteristics**	
Women/Men	71 (67%)/36 (33%)
Age of onset, P50 (P25–P75)	40.7 (28.4–56.3)
Age at diagnosis, P50 (P25–P75)	40.6 (30.2–53.8)
Start-diagnosis years, P50 (P25–P75)	0.13 (0.06–0.44)
**Fulfillment of Criteria Diagnosis**	
Yamaguchi	91 (85%)
Fautrel	58 (54.2%)
Cush	63 (58.9%)
**Baseline characteristics**	
Fever	98 (100%)
Typical exanthema	81 (82.6%)
Constitutional syndrome	44 (44.4%)
Morning stiffness	60 (64.5%)
Arthralgia	101 (99%)
Arthritis	71 (68.9%)
Persistent arthritis	34 (43%)
Odynophagia	80 (78.4%)
Splenomegaly	27 (28.4%)
Hepatic involvement	28 (29.5%)
Hepatosplenomegaly	37 (38.9%)
Lymphadenopathy	43 (43.4%)
Serositis	30 (32.3%)
**Laboratory data**	
ESR ≥ 30 mm/h (*n* = 302)	90 (92.8%)
Ferritin ≥ 1500 (ng/dL)	52 (56.5%)
Ferritin values (mean ± SD), ng/dL	6053 ± 9779
Hemoglobin < 12 g/dL	39 (37.1%)
Leukocytes ≥ 15,000/mm^3^	45 (43.7%)
Platelets ≥ 400,000/mm^3^	26 (29.5%)
ALT ≥ 40 U/L	52 (57.8%)
AST ≥ 40 U/L	50 (58.1%)
GGT ≥ 40 U/L	61 (73.5%)
**Treatments**	
Need of glucocorticoids	63 (63.6%)
Need of biologics	57 (58.8%)
**Type of evolution**	
Mono-episodic	29 (27.4%)
Poly-episodic	29 (27.4%)
Persistent	48 (45.3%)

Data represent *n* (%), except where other statistics are specified. * The results (percentages) for each variable were calculated considering only the number of patients for whom the data were documented.

**Table 2:** Frequency of atypical or non-classical complications among our cohort of 107 patients with adult-onset Still’s disease.

	Prevalence	95% Confidence Interval
Macrophage activation syndrome (missing data = 2)	10 (9.5%)	4.57 to 17.51
Atypical skin lesions (missing data = 9)	38 (38.8%)	27.44 to 53.22
Cardiac complications (missing data = 10)	22 (22.7%)	14.21 to 34.34
Pericardial disease	18 (18.5%)	11.0 to 29.33
Myocarditis	4 (4.1%)	1.12 to 10.56
Suspected pulmonary arterial hypertension	6 (6.2%)	2.27 to 13.46
Inflammatory valvular involvement	1 (1%)	0.03 to 5.74
Primary respiratory disease (missing data = 10)		
Pleural disease	28 (28.9%)	19.18 to 41.72
Transient pulmonary infiltrates	4 (4%)	1.09 to 10.24
Headache (missing data = 8)	13 (14.1%)	7.52 to 24.16
Peritonitis (missing data = 0)	9 (8.4%)	3.85 to 15.97
Secondary amyloidosis (missing data = 3)	1 (0.9%)	0.02 to 5.36

The results are presented as the number of cases and percentages. Their 95% confidence intervals (CIs) were calculated assuming a Poisson distribution.

**Table 3:** The frequency of sequelae or chronic damage attributable to the disease or its treatment.

	Prevalence	95% Confidence Interval
Chronic damage	15 (14.1%)	0.07 to 0.20
Functional impairment	4 (4%)	0.009 to 0.07
Secondary amyloidosis	1 (1%)	0.0 to 0.02
Stunting *	1 (1%)	0.0 to 0.02
Avascular necrosis	1 (1%)	0.0 to 0.02
Cataracts	5 (4.8%)	0.009 to 0.09
Osteoporotic fracture	5 (4.8%)	0.009 to 0.09
Depression	7 (6.7%)	0.01 to 0.11

* The patient’s age at disease onset was 18.2 years. The results are presented as the number of cases and percentages. Their 95% confidence intervals (CIs) were calculated assuming a Poisson distribution.

**Table 4:** The results of the comparative study between patients with and without non-classical clinical manifestations.

	Without Complications (*n* = 43)	With Complications (*n* = 64)	*p*-Value
Fever	34 (100%)	64 (100%)	-
Typical exanthema	22 (64.7%)	32 (50.0%)	0.164
Splenomegaly	8 (25.8%)	19 (29.7%)	0.694
Hepatomegaly	7 (22.6%)	21 (32.8%)	0.305
Odynophagia	27 (71.0%)	53 (82.8%)	0.163
Lymphadenopathy	10 (27.8%)	33 (52.4%)	0.018
Arthritis	27 (69.2%)	44 (68.7%)	0.959
Shoulder involvement	-	2 (22.2%)	1.000
Type of evolution			0.112
Mono-episodic	11 (26.2%)	18 (28.1%)	
Poly-episodic	16 (38.1%)	13 (20.3%)	
Persistent	15 (35.7%)	33 (51.6%)	
Need for high doses of GC	30 (55.6%)	43 (68.2%)	0.206
Need for biologic therapy	30 (55.6%)	37 (60.7%)	0.622
Age at onset	39.4 ± 16.6	44.2 ± 17.8	0.256
ESR (mm/h)	80.4 ± 26.4	73.9 ± 30.8	0.281
CRP (mg/dL)	25.4 ± 36.9	34.1 ± 61.3	0.776
Ferritin values (ng/dL)	4879 ± 6797	6741 ± 11,164	0.955
Hemoglobin (g/dL)	11.1 ± 1.6	11.5 ± 1.7	0.212
Leucocytes/mm^3^	14,118 ± 6177	14,286 ± 5905	0.836
ALT	69.1 ± 89.6	84.9 ± 122.7	0.942
AST	77 ± 65.7	83.3 ± 121.9	0.440
GGT	103.7 ± 87.2	163 ± 195.7	0.428
SSC index	5.3 ± 1.5	6.6 ± 1.7	0.0002

The results are presented as the mean ± standard deviation (SD) or the number of cases with frequencies. The percentages in each variable were calculated considering only the number of patients for whom the data were documented. SSC = systemic score system.

**Table 5:** Predictive variables of development of non-classical clinical manifestations.

	Bivariate OR [95% CI] (*p*-Value)	Multivariate OR [95% CI] (*p*-Value)
Age at onset	1.02 [0.99–1.04] (0.190)	
Female gender	0.71 [0.31–1.65] (0.431)	
Typical exanthema	0.54 [0.23–1.28] (0.166)	0.32 [0.11–0.95] (0.041)
Odynophagia	1.96 [0.75–5.10] (0.167)	
Lymphadenopathy	2.86 [1.18–6.90] (0.019)	2.85 (1.03—7.91] (0.044)
Type of evolution		
• Mono-episodic	1	
• Poly-episodic	0.50 [0.17–1.42] (0.190)	
• Persistent	1.34 [0.51–3.54] (0.549)	
Ferritin values (ng/dL)	1.00 [0.99–1.00] (0.383)	
Hemoglobin (g/dL)	1.13 [0.89–1.43] (0.306)	1.32 [0.96–1.79] (0.087)
SSC index	1.61 [1.22–2.12] (0.001)	1.86 [1.29–2.69] (0.001)
Need for high doses of GC	1.72 [0.74–4.00] (0.208)	
Need for biologic therapy	1.23 [0.53–2.84] (0.622)	

## Data Availability

The authors confirm that all data underlying the findings are fully available without restriction. All relevant data are included in the paper.

## Other Members of the JIA-Still SERPE Register

Agustín Remesal, Hospital Universitario La Paz, Madrid.

Alina Botenau, Hospital Ramón y Cajal, Madrid.

Beatriz Bravo, Hospital Virgen de las Nieves, Granada.

Borja de Miguel Campo, Hospital Universitario 12 de Octubre, Madrid.

Daniel Clemente Garulo, Hospital Niño Jesús, Madrid.

Esmeralda Nuñez, Hospital Materno Infantil de Málaga, Málaga.

Gisela Díaz-Cordovés Rego, Hospital Universitario Carlos Haya, Málaga.

Jaime de Inocencio, Hospital Universitario 12 de Octubre, Madrid.

Jordi Fiter, Hospital Son Espases, Palma de Mallorca.

Jose Alfredo Gómez-Puerta, Hospital Clinic, Barcelona.

Julián Fernández Martín, Hospital Álvaro Cunqueiro, Vigo.

Laura Casas, HUN Sra de Candelaria, Tenerife

Mª del Pilar Hernández Jiménez, Hospital Universitario 12 de Octubre, Madrid.

Mª Sagrario Bustabad Reyes, Hospital Universitario de Canarias, Tenerife.

María del Carmen Pinedo, Hospital Universitario de Cruces, Bilbao.

María Teresa Silva Díaz, Hospital Universitario de La Coruña, Coruña.

Marta Medrano San Ildefonso, Hospital Universitario Miguel Servet, Zaragoza.
